# Functional connectivity changes during working memory in autism spectrum disorder: A two-year longitudinal MEG study

**DOI:** 10.1016/j.nicl.2023.103364

**Published:** 2023-03-02

**Authors:** Julie Sato, Kristina Safar, Vanessa M. Vogan, Margot J. Taylor

**Affiliations:** a Department of Diagnostic Imaging, The Hospital for Sick Children, Toronto, ON, Canada; b Neuroscience & Mental Health Program, The Hospital for Sick Children Research Institute, Toronto, ON, Canada; c Department of Applied Psychology and Human Development, Ontario Institute for Studies in Education, Toronto, ON, Canada; d Department of Medical Imaging, University of Toronto, Toronto, ON, Canada; e Department of Psychology, University of Toronto, Toronto, ON, Canada

**Keywords:** Autism spectrum disorder, Working memory, Functional connectivity, Longitudinal, Children, magnetoencephalography (MEG), ASD, Autism spectrum disorder, MEG, magnetoencephalography, TD, Typically developing, dlPFC, dorsolateral prefrontal cortex, IPL, inferior parietal lobe, ADOS-G, Autism Diagnostic Observation Schedule-General, ADOS-2, Autism Diagnostic Observation Schedule-Second Edition, FSIQ, Full-scale IQ, WASI, Wechsler Abbreviated Scale of Intelligence, WMTB-C, Working Memory Test Battery for Children, BRIEF, Behavior Rating Inventory of Executive Function, SRS-2, Social Responsiveness Scale, Second Edition, AAL, Automated Anatomical Labeling, PLI, phase-lag index, ANOVA, analysis of variance, NBS, Network Based Statistic, FWER, family-wise error rate

## Abstract

Working memory impairments have been reported in adults with autism spectrum disorder (ASD) and associated with functional outcomes and social difficulties. However, little is known about the developmental trajectory of working memory in youth with ASD. The current magnetoencephalography (MEG) study is the first to examine the longitudinal development over two years of working memory networks in youth with ASD. We analysed MEG data from 32 children and adolescents with and without ASD (64 datasets; 7–14 years), all tested twice at a two-year interval, during a visual *n*-back task, with two loads (1- and 2-back). We performed a whole-brain functional connectivity analysis to examine the networks during the successful recognition of visual stimuli. We demonstrate that youth with ASD show decreased connectivity in the theta frequency (4–7 Hz) in the higher memory load (2-back) condition compared to typically developing (TD) controls. This hypo-connected theta network was anchored in primary visual areas with connections to frontal, parietal and limbic regions. These network differences were found despite similar task performance between ASD and TD groups. Within the TD group, we found an increase in alpha (8–14 Hz) connectivity at Time 2 compared to Time 1 in both the 1- and 2-back conditions. These findings demonstrate the continued development of working memory mechanisms over middle childhood, which were not apparent in youth with ASD. Together, our findings support a network-based approach to understanding atypical neural functioning in ASD and the developmental trajectories of working memory processes over middle childhood.

## Highlights

• Longitudinal changes in working memory networks were investigated over two years.
• Youth with autism spectrum disorder (ASD) showed reduced theta (4–7 Hz) connectivity.
• Typically developing youth showed greater alpha (8–14 Hz) connectivity at follow-up.
• These findings demonstrate the maturation of working memory over middle childhood.
• These longitudinal changes were not apparent in children and adolescents with ASD.

## 1 Introduction

Autism spectrum disorder (ASD), or autism, is a pervasive neurodevelopmental disorder characterized by impairments in social communication, as well as restricted and repetitive behaviours (American Psychiatric Association, 2013). However, individuals with ASD can also exhibit a range of cognitive deficits, including working memory impairments (Barendse et al., 2013, Habib et al., 2019). Working memory is the ability to temporarily maintain and manipulate information in mind to guide behaviour (Baddeley and Hitch, 1974, D'Esposito, 2007), and is not only fundamental to academic achievement (Alloway, 2009), but also plays a critical role in social cognition and interpersonal interactions (Dennis et al., 2009, Phillips et al., 2008). Behavioural studies support this link, showing that working memory deficits, most prominent in the visual and spatial domains (Chien et al., 2015, Wang et al., 2017), are associated with developmental outcomes and social functioning in autism (Kercood et al., 2014, Leung et al., 2016). Despite this, few studies have explored the development of cognitive and executive functions in autism, and even fewer have investigated the functional neural correlates underlying working memory processing.

Successful working memory relies on a network of brain regions, including frontal (e.g., dorsolateral prefrontal cortex [dlPFC]), parietal (e.g., inferior parietal lobe [IPL]) and temporal (e.g., inferior temporal gyrus) cortices, to support the maintenance and retrieval of information (Daume et al., 2017, Eriksson et al., 2015, Gazzaley et al., 2004). Most neuroimaging studies examining the functional networks underlying working memory in autism have been conducted with adults (Audrain et al., 2020, Koshino et al., 2005, Luna et al., 2002, Yuk et al., 2020) and demonstrate widespread network alterations consistent with the view that autism is a neural network disorder (Uddin et al., 2013). A recent magnetoencephalography (MEG) study found that adults with ASD exhibited decreased theta-band (4–7 Hz) connectivity in a network involving key fronto-parietal regions compared to control adults (Yuk et al., 2020). These group differences were found during the successful recognition of repeated visual stimuli of a 1-back working memory task; the authors suggested that decreased theta-band connectivity may reflect the inability to supress interference from other stimuli during working memory retrieval.

Developmental neuroimaging studies on working memory functions in ASD are limited. To our knowledge, only one other MEG study assessed connectivity during a working memory task in children with ASD (7–11 years; Urbain et al., 2016). The authors found reduced alpha-band (8–14 Hz) connectivity in a fronto-temporal network in ASD compared to typically developing (TD) children. Importantly, lower connectivity strength in the right fusiform gyrus, a major hub in this hypo-connected network, was significantly correlated with symptom severity (assessed through the Autism Diagnostic Observation Schedule-General [ADOS-G] Lord et al., 2000) in children with ASD. Thus, reduced alpha connectivity strength was associated with increased ASD symptomology. Given the ongoing maturation of the underlying brain structures supporting working memory processes during childhood and adolescence, it is crucial to better understand the neural bases of working memory development in ASD to improve cognitive and behavioural outcomes in this population.

A recent functional MRI study was the first to examine the longitudinal changes (over a 2-year period) in brain activity during a visuo-spatial 1-back task with four levels of difficulty in children with ASD compared to TD controls (7–13 years; Vogan et al., 2019). The authors found that children with ASD showed little age-related change in load-dependent activation, suggesting inadequate modulation of fronto-parietal regions with increasing cognitive load. In contrast, TD children showed increases over time in load-dependent activation in frontal, parietal and occipital areas (Vogan et al., 2019). Thus, despite similar behavioural performance at baseline and follow-up (2 years later), children with ASD showed different developmental trajectories (group × time interaction) of brain activity. Our study will be the first to extend these longitudinal findings using MEG during an *n*-back visual working memory task, with two loads (1- and 2-back), in youth with and without ASD. We investigated the neural correlates of successful recognition of visual stimuli longitudinally (over two years) to examine functional connectivity changes over time. Based on the findings from Vogan et al. (2019), we expected to find functional connectivity changes most prominently in the 2-back memory load condition in children with ASD compared to TD controls. We further expected to find these changes in the frequency bands of theta and alpha, as these have been most frequently implicated in memory processes (Klimesch et al., 2008, Palva et al., 2010, Sauseng et al., 2009).

## 2 Methods

### 2.1 Participants

Sixty-three children and adolescents (38 ASD, 25 TD, 7–14 years) were recruited for this neuroimaging study at the Hospital for Sick Children (SickKids), between 2011 and 2013 (Vogan et al., 2019). All participants were invited back two years later (9–16 years) for a follow-up study. Of the 63 participants, 18 (12 ASD, 7 TD) did not return for the follow-up study due to relocation, declined to participate, had contraindications for MEG (e.g., braces), or were lost to follow-up. MEG data from 13 additional participants (10 ASD and 3 TD) were excluded from analyses due to a) sex matching; b) <20 clean MEG trials; and c) <55% task accuracy. Thus, the final sample consisted of 64 datasets from 17 children with ASD and 15 age- and sex-matched TD controls. The final sample differed slightly for the 2-back memory load condition due to increased task difficulty (58 datasets: 15 ASD, 14 TD). Importantly, as previously reported by Vogan et al. (2019), the participants that returned at follow-up did not significantly differ from those who did not return in terms of age, sex, and IQ. The study protocol was approved by the Research Ethics Board at SickKids. Written informed consent was obtained by a parent or legal guardian, and informed verbal assent was provided by all children. For TD controls, exclusion criteria included a diagnosis of a learning, language or neurodevelopmental disorder; for both groups exclusion criteria also included history of prematurity, severe neurological damage, uncorrected visual impairment or colour blindness and IQ < 70. For children in the ASD group, a primary diagnosis of ASD was confirmed by the Autism Diagnostic Observation Schedule-Second Edition (ADOS-2; Lord et al., 2012) by expert clinicians. A summary of the demographic characteristics is shown in Table 1.

**Table 1:** Participant demographics.

	Time point	ASD (n = 17)	TD (n = 15)	Significance test
		*M* (SD) or count	*M* (SD) or count	
Sex (M:F)	Baseline	15:2	8:7	*p =* 0.05†
Age (years)	Baseline Follow-up	11.13 (1.83) 13.50 (1.58)	10.69 (2.32) 12.91 (2.29)	*t*(30) = 0.59, *p* = 0.56 *t*(30) = 0.85, *p* = 0.40
ADOS-2	Baseline Follow-up	6.29 (2.05) 7.13 (2.28)	–	–

†A Fisher’s exact test was used to test for differences in the proportion of boys and girls between-groups.

Full-scale IQ (FSIQ) was measured using the two sub-test version of the Wechsler Abbreviated Scale of Intelligence (WASI; Wechsler, 2013) for all children at both time points. FSIQ scores were estimated based on performance on the Vocabulary and Matrix reasoning sub-tests. To assess working memory ability, two sub-tests of the Working Memory Test Battery for Children (WMTB-C) (Gathercole & Pickering, 2000) were administered (Digit Recall and Block Recall). Parents also completed questionnaires on executive function abilities and social impairment using the Behavior Rating Inventory of Executive Function (BRIEF; Gioia et al., 2000) and the Social Responsiveness Scale, Second Edition (SRS-2; Constantino, 2012), respectively.

### 2.2 Working memory *n*-back task

**Fig. 1:**
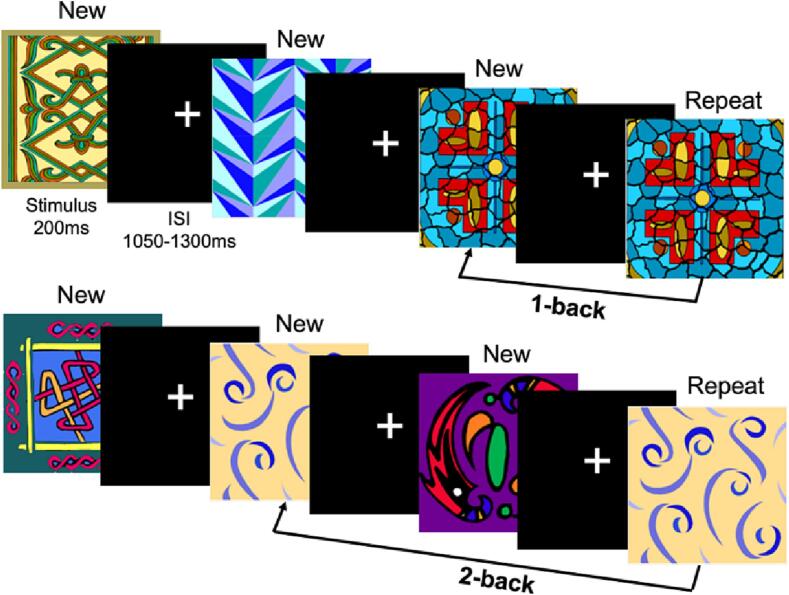
**The *n*-back task.** Participants pressed a button as rapidly as possible when they recognized that an image was the same (Repeat) as the image presented *n* trials earlier (New). The top panel represents the 1-back condition, and the bottom panel represents the 2-back condition.

Children performed a visual *n-*back task in the MEG with two loads, 1- and 2-back (Fig. 1) in separate blocks, which were counterbalanced across participants. The task stimuli consisted of multi-coloured patterned images that are presented for a duration of 200 ms, with an inter-stimulus interval (fixation cross) jittered between 1050 and 1300 ms. Children identified, as quickly as possible, the repeat of an identical image presented *n* trials previously by button-press. The 1-back task included 190 New trials (unique images presented for the first time) and 95 Repeat trials (images presented for the second time). The 2-back task included 221 New and 109 Repeat trials. The task was run using Presentation® software (www.neurobs.com); the images subtended ∼4° of visual angle, from a viewing distance of 80 cm. Recognition accuracy and reaction time for Repeat trials were recorded for behavioural analyses. Data were included in MEG analyses for participants with >55% recognition accuracy for Repeat trials; only correct Repeat trials were analyzed.

### 2.3 MEG acquisition

MEG data were recorded using a 151-channel CTF system (CTF MEG International Services LP, Coquitlam, Canada) in a magnetically shielded room, while participants lay in the supine position. Data were collected at a sampling rate of 600 Hz with an online antialiasing filter (0–150 Hz) and a third-order spatial gradient to attenuate background noise. Participants were fitted with fiducial coils placed at the nasion and left and right pre-auricular areas to continuously track head movement throughout the recording. Following MEG recording, the fiducial coils were substituted with radio-opaque markers for MRI co-registration. Individual T1-weighted MR images were collected in all participants using a Siemens 3 T MAGNETOM Trio with a 12-channel head coil (TR/TE = 2300/2.96 ms, FA = 9°, FOV = 240x256mm, # slices = 192, resolution = 1.0 mm isotropic) scanner for MRI co-registration with the MEG data.

### 2.4 MEG data pre-processing and source reconstruction

MEG data pre-processing and source reconstruction were performed using the FieldTrip toolbox (Oostenveld et al., 2011) in MATLAB (The Mathworks Inc, 2018). A 4th order two-pass Butterworth filter was used to filter the data between 1 and 150 Hz; line noise was eliminated from the signal using a discrete Fourier transform notch filter at 60 Hz and 120 Hz. The data were then epoched from −500 to 600 ms relative to the onset of the Repeat image. To attenuate signal artefacts (e.g., ocular and cardiac), independent component analysis (ICA; ‘fastica’ function) was performed and identified components were removed manually. Following ICA, trials were excluded from analyses if: a) the signal exceeded +/-2000ft, b) initial median head position shifted >10 mm (recommended in Pang (2011)). Head motion for the 1-back load condition did not significantly differ between the groups *F*(1, 30) = 0.31, *p* = 0.579, or time *F*(1, 30) = 0.27, *p* = 0.606, nor was there a group-by-time interaction *F*(1, 30) = 2.40, *p* = 0.131. We did not find any significant difference in head motion between groups nor group-by-time interaction for the 2-back condition (*p* > 0.05). Data from participants with>20 clean trials remaining after artefact rejection were included in subsequent analyses. For the 1-back, there were no significant difference in the number of trials included between groups *F*(1, 30) = 1.53, *p* = 0.225, or time *F*(1, 30) = 0.06, *p* = 0.802, nor was there a group-by-time interaction *F*(1, 30) = 0.03, *p* = 0.868. No significant group difference in the number of trials nor group-by-time interaction was found for the 2-back condition (*p* > 0.05).

Individual T1-weighted MRIs were co-registered to the participants’ MEG data. A single shell head model was generated (Nolte, 2003) for each child based on his or her anatomical MRI using SPM12 through FieldTrip. The centre-of-mass of the first 90 coordinates of the Automated Anatomical Labelling (AAL) atlas (Tzourio-Mazoyer et al., 2002) were non-linearly transformed onto equivalent subject-specific coordinates from standard template space (ICBM 152; (Fonov et al., 2011). Linearly constrained minimum variance beamforming was used to estimate the broadband timeseries at each of the 90 source locations (van Veen et al., 1997), with 5% Tikonov regularization. The neural activity index was estimated to account for the centre-of-head bias due to correlated noise (van Veen et al., 1997).

### 2.5 Functional connectivity: Phase lag index

The source reconstructed broadband time series data at each of the AAL parcels were filtered into frequency bands: theta (4–7 Hz), alpha (8–14 Hz), beta (15–29 Hz) and gamma (30–55 Hz). The Hilbert Transform was used to generate instantaneous phase values at each sample across the timeseries at each source and frequency band. Instantaneous phase synchrony between pair-wise sources was then calculated using the cross-trial phase-lag index (PLI; Stam et al., 2007), yielding a 90-by-90 adjacency matrix for each frequency band and every subject. A time window of 0 to 300 ms has been shown to be sensitive to detecting group differences in functional connectivity during working memory recognition between ASD and TD controls (Urbain et al., 2016, Yuk et al., 2020); a baseline window of −300 to 0 ms relative to stimulus onset was selected. The PLI values in the active window were z-scored relative to the baseline window and then averaged.

### 2.6 Statistics

For behavioural accuracy and reaction time data, a mixed analysis of variance (ANOVA) design was used to examine differences between group (ASD, TD), time (Time 1, Time 2), and a group-by-time interaction. The Network Based Statistic (NBS; (Zalesky et al., 2010, Zalesky et al., 2012)) was used to analyze functional connectivity in the MEG data. A mixed ANOVA was performed for each frequency band and was used to test for a main effect of group and time, as well as group-by-time interaction for both load conditions. To ensure that the between-group network results did not differ based on sex, we also ran a group-by-sex interaction. Within-group differences, between Time 1 and Time 2, were also investigated. Lastly, we examined the interaction between group-by-memory load. The NBS is a widely used non-parametric statistical tool to identify possible networks related to an experimental effect, while controlling for the family-wise error rate (FWER) (Zalesky et al., 2010, Zalesky et al., 2012). The primary component-forming thresholds for analyses were determined based on 1% of total possible network connections (∼40 connections); 5,000 permutations were performed. Pearson’s correlations were also performed between mean network connectivity strength in the resulting significant networks with behavioural measures (e.g., task accuracy, WMTB-C, BRIEF-Working Memory, SRS-2). Region-specific connectivity strength was calculated by taking the sum of the PLI values at each region to all other brain regions in the significant network; the mean across these values were then computed resulting in the mean connectivity strength for each participant.

## 3 Results

### 3.1 Participant characteristics

FSIQ was significantly higher in the TD than the ASD group, however, there were no main effects of time (see Supplemental Table 1 for summary statistics). For the Digit Recall of the WMTB-C, the TD group scored significantly higher than the ASD group, but no main effect of time was found (Supplemental Table 1). For Block Recall, no main effects of group or time were found. For the BRIEF, children with ASD had significantly greater working memory problems compared to their TD peers; no main effect of time was found. Average T-scores for the BRIEF working memory scale were in the potentially clinically elevated range (65 to 69) for the ASD group. For the SRS-2, the ASD group showed significantly greater social impairment compared to the TD group. We also found a main effect of time, with scores improving in both groups at Time 2. Average SRS-2 total scores were in the moderate (66 to 75) to severe (≥76) risk category in children with ASD. In the MEG scanner, children performed the 1- and 2-back load conditions in separate blocks. For both load conditions, no main effects of group or time were found. There were no significant group-by-time interactions found for any MEG task behavioural measures. See Table 2 for a summary of behavioural performance.

**Table 2:** Summary of behavioural performance.

		**ASD (n = 17)**	**TD (n = 15)**
		*Mean* (SD)	*Mean* (SD)
Full-scale IQ	Baseline Follow-up	104.6 (13.5) 103.3 (14.3)	119.7 (11.1) 116.0 (9.1)
WMTB-C Digit recall Block recall †	Baseline Follow-up Baseline Follow-up	103.0 (21.5) 101.1 (24.5) 92.8 (15.8) 88.4 (15.0)	119.1 (16.8) 120.8 (15.0) 100.8 (23.6) 102.6 (20.5)
BRIEF-Working Memory	Baseline Follow-up	67.7 (8.1) 66.8 (9.7)	47.8 (11.3) 45.9 (10.8)
SRS-2 Total score	Baseline Follow-up	76.1 (13.2) 72.0 (14.0)	45.9 (8.9) 44.5 (6.3)
1-back accuracy (%)	Baseline Follow-up	86.7 (14.3) 89.6 (11.9)	94.2 (8.0) 94.6 (7.1)
2-back accuracy (%)‡	Baseline Follow-up	66.6 (14.1) 67.6 (15.0)	69.0 (17.2) 74.0 (15.5)
1-back, trials included in the analysis, n	Baseline Follow-up	56.9 (16.4) 58.6 (17.5)	62.6 (13.3) 62.9 (15.7)
2-back, trials included in the analysis, n	Baseline Follow-up	67.2 (16.7) 61.9 (18.4)	61.6 (24.4) 69 (22.7)

†16 ASD and 14 TD children completed the block recall at Time 1 and 16 ASD and 15 TD children at Time 2. ‡15 ASD, 14 TD were included in the 2-back analyses due to poor task performance.

### 3.2 Between-group MEG network analyses

**Fig. 2:**
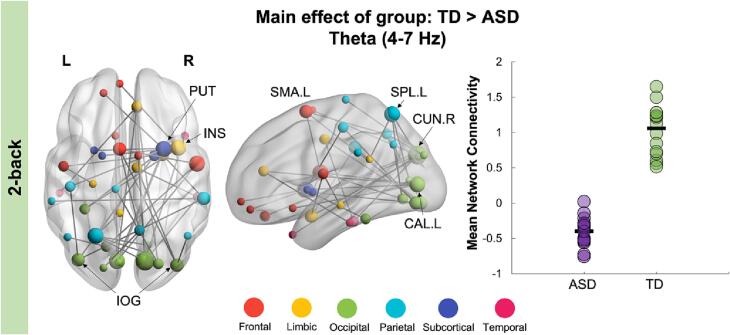
**Significant network for the main effect of group on functional connectivity, 0**–**300 ms following the repeat trial onset (i.e., recognition window) in the 2-back load.** The TD group showed increased connectivity in the theta-band compared to children with ASD. The node sizes in the brain network (left image) are scaled by degree. Network hub regions are labelled on the brain plot. The mean network connectivity strength was extracted from this network and plotted on the right-side bar graph.

Using NBS, we performed a mixed ANOVA in the 1- and 2-back load conditions. There were no main effects of group or time, nor significant group-by-time interaction in the 1-back condition (all *p_corr_* > 0.05). We did, however, find a main effect of group in the 2-back condition, such that the ASD group showed decreased connectivity in the theta frequency band compared to the TD group during successful recognition of visual stimuli (40 edges, 40 nodes, *p_corr_* = 0.005; Fig. 2). This network was anchored in occipital regions, including the inferior occipital gyrus, right cuneus and left calcarine. In addition, network hubs were found in parietal (left superior parietal lobe), limbic (right insula) and frontal (left supplementary motor area) areas. No main effects of time were found, nor any significant group-by-time or group-by-sex interactions (all *p_corr_* > 0.05). We also analyzed the group-by-load interaction at both time points to determine if groups showed differences in load-dependent functional connectivity. No significant interaction effects were found (all *p_corr_* > 0.05).

### 3.3 Within-group MEG network analyses

**Fig. 3:**
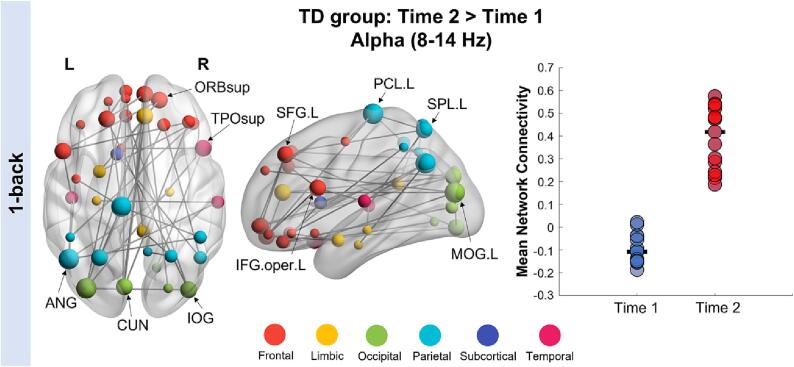
**Network of regions showing increased alpha connectivity in the 1-back load at Time 2 in the TD group, occurring between 0** and **300 ms, post repeat trial onset.** TD children showed significantly increased connectivity at Time 2 compared to Time 1 in this alpha-band network. Node size is scaled by degree (left image) and mean connectivity strength for this network at each time point is plotted on the right-side bar graph. Network hub regions are labelled on the brain plot.

**Fig. 4:**
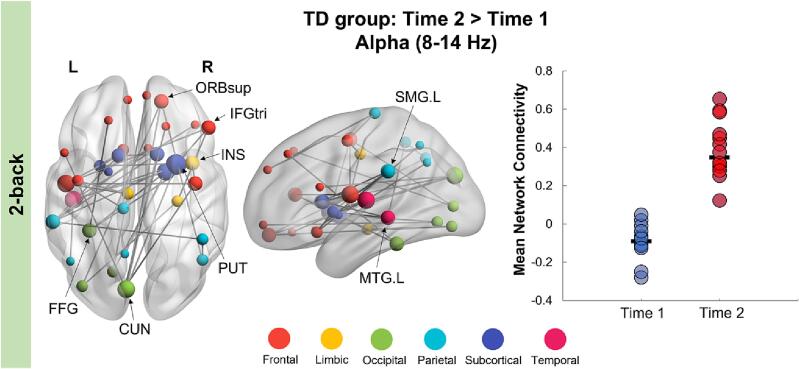
**Network of regions showing increased alpha connectivity in the 2-back load at Time 2 in the TD group, occurring between 0** and **300 ms, post repeat trial onset.** TD children showed significantly increased alpha connectivity at Time 2 compared to Time 1. The extracted mean connectivity strength of this network is plotted on the right-side bar graph. Network hub regions are labelled on the brain plot.

We also investigated within-group functional connectivity changes over time in both groups. In the TD group, we found significantly increased connectivity at Time 2 compared to Time 1 in the alpha frequency band (40 edges, 38 nodes, *p_corr_* = 0.026; Fig. 3). This distributed alpha-band network had major hubs in the frontal (left superior frontal gyrus, right superior orbitofrontal gyrus, left inferior frontal gyrus - pars opercularis), parietal (left superior parietal lobe, angular gyrus and paracentral lobule) and occipital (left middle occipital gyrus, left cuneus and right inferior occipital gyrus) regions. For the 2-back load, TD children also showed significantly increased alpha-band connectivity at Time 2 (40 edges, 35 nodes, *p_corr_* = 0.013; Fig. 4). The node with the highest degree in this network was the right putamen, with other network hubs found in the frontal (right superior orbital frontal gyrus and inferior frontal gyrus - pars triangularis), limbic (right insula), subcortical (bilateral caudate), occipital (left cuneus) and temporal (left fusiform and middle temporal gyrus) areas. No significant networks were found in the ASD group between Time 1 and Time 2 in either the 1- or 2-back loads. We also investigated brain-behaviour correlations within-group, between the mean connectivity strength of significant networks with working memory performance and social functioning. We found no significant associations with any behavioural measures in either the TD or ASD groups.

## 4 Discussion

Our study is the first to use MEG to examine the longitudinal development over two years of working memory networks in youth with and without ASD. We demonstrate that children with ASD show decreased connectivity in the theta frequency compared to TD controls during successful recognition of visual stimuli. This between-group difference was found across time, in the higher memory load condition (2-back). This hypo-connected network was anchored in primary visual areas with connections to frontal, parietal and limbic regions. Consistent with previous neuroimaging studies, these functional connectivity differences were found despite similar task performance between ASD and TD groups. However, parent-reported measures of working memory difficulties were significantly higher in children with ASD at both time points. We also investigated functional connectivity changes over time within each group. We found no significant effects in the ASD group, while TD children showed a significant increase in alpha-band connectivity at Time 2 compared to Time 1 in both the 1- and 2-back conditions. These findings demonstrate the continued development of working memory mechanisms over middle childhood, which were not apparent in children and adolescents with ASD.

Our between-group network findings of decreased theta-band connectivity in children with ASD was confined to the 2-back condition, consistent with other studies reporting working memory deficits when a heavier cognitive load was imposed (Barendse et al., 2013). Previous work in youth have reported atypical activation and connectivity within the classic frontoparietal working memory network, involving the dlPFC and IPL (Urbain et al., 2015, Barendse et al., 2018). In our study, however, we found hypo-connectivity in a posteriorly anchored network with major hubs in the left superior parietal lobe, right insula, right cuneus and left calcarine in children with ASD. Somewhat unexpected was the lack of frontal connections in this between-group network, which may be attributable to the relatively high-functioning sample of ASD youth included in this study; evident by the similar performance in both the 1- and 2-back conditions. However, the involvement of the left superior parietal lobe and right insula, both regions implicated in executive control processes, is reflective of the higher task demands of the 2-back load. For instance, the superior parietal lobe is involved in the manipulation and updating of information within working memory (Koenigs et al., 2009), which is critical to successful performance in the more demanding 2-back load, requiring constant monitoring of visual items. In addition, the anterior insula, and the adjacent inferior frontal gyrus, are part of the frontoparietal network subserving working memory (Mencarelli et al., 2019, Rottschy et al., 2012), especially during more challenging task conditions (Eckert et al., 2009). Thus, reduced theta connectivity found in the ASD group may reflect the use of a compensatory network to support similar task performance. In addition, these findings may also indicate a vulnerability to sustain working memory in more challenging real-world conditions that require one to rapidly adapt to changing social cues and increasing demands. This is further supported by the fact that parent-reported measures of social impairment and working memory difficulties were significantly higher in children and adolescents with ASD.

Theta oscillations are known to play an important role in top-down control (von Stein et al., 2000, Sauseng et al., 2010), as well as working memory encoding and retrieval/recognition processes (Klimesch et al., 2001, Sauseng et al., 2004). A prior MEG cross-sectional study found reduced connectivity in the 2-back condition in the alpha-band during working memory recognition in children (7–13 years) with ASD (Urbain et al., 2016). This discrepancy may be related to differences in the age-range of participants, as our study included two time points spanning children and adolescents aged 7 to 16 years. Consistent with this, our findings are similar to an MEG study in young adults with ASD showing decreased theta-band connectivity in a frontoparietal network (Yuk et al., 2020). These findings suggest that atypical theta connectivity underlying working memory may persist into adulthood in individuals with ASD and contribute to broader difficulties in social functioning (Larrain-Valenzuela et al., 2017). Given the critical role of theta oscillations in mediating long-range neural communication, our findings emphasize the developmental perspective of long-range underconnectivity in adolescents and adults with ASD reported in fMRI studies (Uddin et al., 2013).

While we did not find significant differences in the developmental trajectories of functional connectivity between-groups, we found that TD children showed increased alpha connectivity at Time 2 compared to Time 1. These findings demonstrate the continued maturation of working memory mechanisms over this dynamic two-year period as children progress into adolescence, which was not present in the ASD group. This increase in alpha-band connectivity at Time 2 was observed for both the 1- and 2-back conditions, emphasizing the important role of alpha oscillations during working memory tasks in typical development (Jensen et al., 2002, Sato et al., 2018). While previous studies have established the role of long-range alpha synchrony during working memory maintenance, our study found that correct recognition of visual stimuli was associated with alpha synchrony, which increased over time. Thus, our data are extending the literature by demonstrating that alpha oscillations are important for mediating inter-regional synchrony for both maintenance and recognition processes over childhood and adolescence (Sato et al., 2018, Doesburg et al., 2010). In the 1-back network, we found major hubs in frontal, parietal and occipital regions including the left-lateralized angular gyrus. While the angular gyrus is a core region of the default mode network, it is also associated with memory retrieval processes (Yeo et al., 2011, Kim, 2010), especially during verbal working memory tasks (Emch, von Bastian, & Koch, 2019). Thus, it is possible that children and adolescents may use verbal strategies to guide their performance during the less demanding 1-back condition. Further, it is important to note that the lack of functional connectivity changes in the ASD group make interpretation about maturation changes difficult. It is also possible that we did not find differences in the developmental trajectories between-groups due to a lack of statistical power. It would be valuable for future, larger studies to investigate changes across different periods of childhood and adolescence to determine whether maturation is delayed and/or altered in ASD.

In the 2-back network, TD controls showed increased alpha connectivity in a network largely anchored in temporal (left middle temporal and fusiform gyri) and subcortical (right putamen, bilateral caudate) regions, with other hubs in frontal regions including the ventrolateral prefrontal cortex known to be involved in working memory (Owen et al., 2005). The putamen and caudate, both components of the basal ganglia, interact closely with the prefrontal cortex to support visual working memory (Voytek & Knight, 2010); i.e., the basal ganglia are involved in the dynamic updating of visual working memory representations that are actively sustained by the prefrontal cortex (Voytek and Knight, 2010, Frank et al., 2001). This is consistent with the higher cognitive demands of the 2-back condition, requiring both maintenance and monitoring of information in working memory. Within the ASD group, however, we did not find any functional connectivity changes over time, which may reflect a delay in the maturation of working memory processes that are supported by alpha oscillations. Longitudinal investigations with a larger sample size are needed to confirm how the developmental trajectories associated with working memory in children with ASD differ from their TD peers.

While previous working memory studies have reported associations between functional connectivity measures in both theta and alpha frequency bands with symptom severity in autism (Urbain et al., 2016, Larrain-Valenzuela et al., 2017), we did not find significant brain-behaviour associations despite having a similar sample size. It is possible that brain processes underlying recognition in working memory are distinct from those related to overall behavioural difficulties observed in autism. It would therefore be important to investigate the functional connectivity differences during the encoding and maintenance phases of working memory to understand the full scope of working memory deficits and their impact on developmental outcomes in autism.

While our study has many strengths including being the first MEG study to report longitudinal effects in children and adolescents with ASD during working memory, there are some limitations to consider. First, our sample size was small, due to a combination of factors, such as participant attrition. Despite using a relatively short follow-up period (2 years), many participants were lost to follow-up or were ineligible due to contraindications for MEG (e.g., braces or dental fillings) at Time 2. Our sample size was further reduced due to exclusion of data that did not pass strict quality control at either time-point. Unfortunately, this data loss is common in longitudinal studies and paediatric neuroimaging; we also acknowledge that our sample may not be representative of all children with ASD, given the known heterogeneity in this group. Further, while we had more youth with ASD who did not participate in the follow-up (n = 12) compared to the TD group (n = 7), there was no significant difference between participants (in the larger cohort) that returned at follow-up in terms of age, sex, and IQ to those who did not return (Vogan et al., 2019). We want to emphasize the tremendous challenges with conducting longitudinal developmental studies, particularly in clinical populations. Our study is one of very few with longitudinal neuroimaging data in children with ASD. Thus, we believe our smaller sample size is reasonable given these challenges and fills a critical gap in the literature. However, future studies with larger sample sizes are encouraged to confirm these findings. Second, while efforts were made to recruit participants matched by sex and age, the longitudinal design, and difficulties with participant attrition, prevented us from being able to match groups optimally on sex. To account for this, we tested for any group by sex interactions to ensure that our between-group network results did not differ based on sex. This interaction was not significant. Third, our sample included a wide age range (7–14 years at baseline); due to difficulties in recruiting children with ASD from the community, as the study visit required almost a full-day visit, placing high demands on family’s schedules. Finally, we did not collect measures of socioeconomic status (SES), which may have provided important supplemental information on the participants, as higher SES can be associated with improved outcomes.

In summary, our study is the first to determine longitudinal changes in neurophysiological functional connectivity associated with working memory recognition in youth with and without ASD. We demonstrate that children and adolescents with ASD show decreased theta-band connectivity compared to TD controls during the higher memory load (2-back) condition. While performance on the *n*-back task was similar between groups, parent-reported impairments in working memory and social functioning were significantly higher in the ASD group. We also found increased alpha-band connectivity at the two-year follow-up in the TD group only, demonstrating the continued development of working memory mechanisms as children progress into adolescence.

## 5 Ethics approval statement

The Hospital for Sick Children’s Research Ethics Board (REB# **1000007826**) approved the study protocol.

## CRediT authorship contribution statement

**Julie Sato:** Conceptualization, Writing – original draft, Investigation, Methodology, Formal analysis, Visualization. **Kristina Safar:** Conceptualization, Writing – original draft, Investigation, Methodology, Formal analysis, Visualization. **Vanessa M. Vogan:** Conceptualization, Investigation, Writing – review & editing. **Margot J. Taylor:** Conceptualization, Supervision, Funding acquisition, Resources, Data curation, Writing – review & editing.

## Declaration of Competing Interest

The authors declare that they have no known competing financial interests or personal relationships that could have appeared to influence the work reported in this paper.

## Data Availability

Data will be made available on request.
