# Supplementary material for: Functional connectivity changes during working memory in autism spectrum disorder: A two-year longitudinal MEG study
Source: Neuroimage Clin. 2023 Mar 2;37:103364. doi: 10.1016/j.nicl.2023.103364 (PMC9999263; doi:10.1016/j.nicl.2023.103364)
Supplement: Supplementary data 1 [file mmc1.docx]

**Functional connectivity changes during working memory in autism spectrum disorder: A two-year longitudinal MEG study**

**Supplemental Table 1**. Behavioural statistics for youth with and without ASD.

| **Assessment** | **Main effect of group** | **Main effect of time** | **Group-by-time interaction** |
| --- | --- | --- | --- |
| Full-scale IQ | *F*(1, 30)=12.29, *p*=0.001 | *F*(1, 30)=2.05, *p*=0.163 | *F*(1, 30)=0.48, *p*=0.493 |
| **WMTB-C**  Digit recall  Block recall | *F*(1, 30)=7.636, *p*=0.01  *F*(1, 27)=2.84, *p*=0.103 | *F*(1, 30)=0.001, *p*=0.972  *F*(1, 27)=0.047, *p*=0.83 | *F*(1, 30)=0.40, *p*=0.532  *F*(1, 27)=1.70, *p*=0.203 |
| BRIEF, Working Memory scale | *F*(1, 30)=39, *p*<0.001 | *F*(1, 30)=1.03, *p*=0.317 | *F*(1, 30)=0.112, *p*=0.740 |
| SRS-2 Total Score | *F*(1, 29)=55.2, *p*<0.001 | *F*(1, 29)=5.627, *p*=0.025 | *F*(1, 29)=1.337, *p*=0.257 |
| 1-back accuracy | *F*(1, 30)=4.127, *p*=0.051 | *F*(1, 30)=0.498, *p*=0.486 | *F*(1, 30)=0.265, *p*=0.61 |
| 2-back accuracy | *F*(1, 27)=0.686, *p*=0.415 | *F*(1, 27)=1.879, *p*=0.182 | *F*(1, 27)=0.816, *p*=0.374 |
